# Neurexin1α knockout in rats causes aberrant social behaviour: relevance for autism and schizophrenia

**DOI:** 10.1007/s00213-024-06559-z

**Published:** 2024-02-29

**Authors:** E. J. Marijke Achterberg, Barbara Biemans, Louk J. M. J. Vanderschuren

**Affiliations:** 1 Department of Population Health Sciences, Section Animals in Science and Society, Faculty of Veterinary Medicine, Utrecht University, Utrecht, the Netherlands; 2 Roche Innovation Center Basel, F. Hoffmann-La Roche Ltd, Basel, Switzerland

**Keywords:** Neurexin1α knockout rats, Social behaviour, Social play, Social motivation, Autism, Schizophrenia

## Abstract

**Rationale:**

Genetic and environmental factors cause neuropsychiatric disorders through complex interactions that are far from understood. Loss-of-function mutations in synaptic proteins like neurexin1α have been linked to autism spectrum disorders (ASD) and schizophrenia (SCZ), both characterised by problems in social behaviour. Childhood social play behaviour is thought to facilitate social development, and lack of social play may precipitate or exacerbate ASD and SCZ.

**Objective:**

To test the hypothesis that an environmental insult acts on top of genetic vulnerability to precipitate psychiatric-like phenotypes. To that aim, social behaviour in neurexin1α knockout rats was assessed, with or without deprivation of juvenile social play. We also tested drugs prescribed in ASD or SCZ to assess the relevance of this dual-hit model for these disorders.

**Results:**

Neurexin1α knockout rats showed an aberrant social phenotype, with high amounts of social play, increased motivation to play, age-inappropriate sexual mounting, and an increase in general activity. Play deprivation subtly altered later social behaviour, but did not affect the phenotype of neurexin1α knockout rats. Risperidone and methylphenidate decreased play behaviour in both wild-type and knockout rats. Amphetamine-induced hyperactivity was exaggerated in neurexin1α knockout rats.

**Conclusion:**

Deletion of the neurexin1α gene in rats causes exaggerated social play, which is not modified by social play deprivation. This phenotype therefore resembles disinhibited behaviour rather than the social withdrawal seen in ASD and SCZ. The neurexin1α knockout rat could be a model for inappropriate or disinhibited social behaviour seen in childhood mental disorders.

## Introduction

Genetics and environmental factors interact in a complex fashion to cause, precipitate or exacerbate mental disorders, but the underlying mechanisms remain elusive. Moreover, only a proportion of individuals that exhibit prodromal signs, such as social withdrawal, ultimately develop true psychiatric symptoms, likely due to this combination of genetic predisposition and environmental factors (Shields 1980). Schizophrenia (SCZ) and autism spectrum disorders (ASD) are neurodevelopmental disorders with strong genetic components, but also characterised by very diverse clinical presentations despite shared genetics. This is most clearly illustrated in monozygotic twins, in which, although concordance rates are higher, differences still exist that can only be explained by (early) life factors that change the course of the disease (Gejman et al. 2010; Imamura et al. 2020). Genetic analyses have revealed a multitude of candidate vulnerability genes, many of which code for synaptic proteins. Of these, the neurexin-1 (nrxn1) gene has been linked directly to ASD and SCZ (Kim et al. 2008; Rujescu et al. 2009, Glessner et al. 2009; Ching et al. 2010; Gauthier et al. 2011). The neurexin gene family consists of three members (neurexin1–3) that encode presynaptic cell adhesion proteins that form trans-synaptic complexes with postsynaptic neuroligins. These neurexin-neuroligin complexes are thought to mediate synaptic signalling, specification and maintenance, and to control the balance of inhibitory GABAergic and excitatory glutamatergic neurotransmission (Südhof 2008). Importantly, alterations in the balance between excitatory and inhibitory signalling in neural micro-circuitry have been implicated in social deficits as seen in ASD and SCZ (Yizhar et al. 2011).

To investigate the causative role of nrxn1 in ASD- and SCZ-like phenotypes, animal models with a genetic ablation have been created. In mice, ablation of nrxn1 resulted in detrimental effects on prepulse inhibition and spatial memory, and increased grooming and motor learning (Etherton et al. 2009; Blundell et al. 2010), but in the social spectrum, only mild phenotypes and varying results have been described (Grayton et al. 2013, Rabaneda et al. 2014, Dachtler et al. 2015; Armstrong et al. 2020). To specifically explore the role of nrxn1 deletion on social behaviour throughout development, rats are preferrable to study because their complex social behavioural repertoire, and especially the characteristic display of play behaviour early in development. Previous studies with nrxn1-deficient rats have shown profound impairments in attention and cognitive capacities, as well as hyperactivity (Esclassan et al. 2015), altered patterns of social interest (Twining et al. 2017), sex-specific deficits in ultrasonic vocalizations and social play, but unaffected sociability and social discrimination (Kight et al. 2021). Janz et al. (2022) reported hyperactivity, deficits in context-dependent auditory processing but a normal response towards social stimuli. Recently, increased social interest, as well as reduced anxiety in the open field, was reported in rats in which the nrxn1 gene was down-regulated in the medial prefrontal cortex (Wu et al. 2023).

Social dysfunction, which has an enormous impact on everyday life, is a hallmark of a ASD and SCZ. These social deficits typically remain untreated, mostly because the underlying neural mechanisms are poorly understood. Importantly, ASD and SCZ manifest already in childhood or adolescence, indicating that the development of the social brain is a process vulnerable to early disturbances. During early life, social interactions typically take the form of social play behaviour, which is abundant in the young of most mammalian species, including rats and humans. Alongside its pleasurable properties, social play is thought to contribute to the development of brain and behaviour (Panksepp et al. 1984; Graham and Burghardt 2010; Vanderschuren and Trezza 2014; Pellis et al. 2023). Indeed, depriving rats of social play behaviour during development results in impairments in the social, emotional and cognitive domains (Vanderschuren and Trezza 2014; Pellis et al. 2023). With regard to human psychopathology, social withdrawal is a prominent negative symptom of SCZ (Marder and Galderisi 2017). A preference for solitary play in childhood seems to predict SCZ (Helgeland and Torgersen 2005; Jones et al. 1994) and play behaviour is disrupted during the prodromal phase of SCZ (Møller and Husby 2000). Furthermore, play has been shown to be impaired in children with ASD (Jarrold 2003; Manning and Wainwright 2010) and impaired social play in ASD is thought to intensify the disorder (Jordan 2003).

To further understand how genetics and the early social environment interact to worsen ASD- or SCZ-like symptomatology, in the present study we used deletion of the neurexin1α gene and lack of social play as the genetic vulnerability and environment insult, respectively. We hypothesize that decreased social interaction the juvenile period acts on top of a genetic vulnerability to precipitate abnormal social behaviour in adolescence and adulthood. Specific aims were: 1. to investigate juvenile, adolescent and adult social (play) behaviour of neurexin1α-knockout (nrxn1-KO) rats; 2. to identify neuropharmacological correlates of the altered behaviour in nrxn1-KO rats, by testing risperidone and methylphenidate, drugs commonly used for symptom management in ASD and SCZ, as well as amphetamine, that exacerbates symptoms of SCZ; 3. to establish a ‘dual-hit’ model for ASD/SCZ, by subjecting nrxn1-KO rats to deprivation of social play behaviour.

## Materials and methods

### Animals and housing conditions

Male and female neurexin1α-knockout (nrxn1-KO) rats and wild type (WT) control rats (Horizon Discovery, Boyertown, PA) were bred in the animal facilities at Utrecht University and the Roche Innovation Center Basel. Rats were homozygous knockouts, containing a bi-allelic deletion of the nrxn1α gene on a Sprague Dawley background. WT and nrxn1-KO rats were bred from heterozygous pairs. Pups were toe-clipped on post-natal day (PND) 7-9 or ear-punched on PND14 for genotypic validation and weaned between PND21-24. Animals were housed with same-genotype and same-sex cagemates in temperature-controlled rooms (20-21°C, 60-65% relative humidity) in Macrolon cages with ad libitum access to food and water, under a 12:12-h light–dark schedule (lights on at 7.00 a.m.). All experiments were performed during the light phase of the day-night cycle. All experiments were approved by the Animal Ethics Committee of Utrecht University and the Kantonal Experimentation Committee and were conducted in accordance with Dutch (Wet op Dierproeven, 1996), Swiss (Federal Act on Animal Protection; 1978) and European legislation (Guideline 86/609/EEC; Directive 2010/63/EU). For an overview of the cohorts of animals and the experiments in which they were used, see Table 1.

**Table 1:** Overview of cohorts of animals used

Cohort	Facility	Animals	Sex	Tests	Figure
1	UU	Male SOC WT: 13	Male, Female	Play (week 4 and 7) Adult social behaviour (week 11) Locomotor activity	1 4, Table 2 4, Table 2 5 6
		Male SOC KO: 11			
		Male PD WT: 13			
		Male PD KO: 12			
		Female SOC WT: 13			
		Female SOC KO: 9			
		Female PD WT: 13			
		Female PD KO: 10			
2	UU	Male SOC WT: 10	Male	Responding for play	2
		Male SOC KO: 10			
3	UU	Male SOC WT: 4	Male, Female	Play (week 4 and 7) Adult social behaviour (week 11) Social interest (3 chamber task)	1 4, Table 2 4, Table 2 5
		Male SOC KO: 6			
		Male PD WT: 6			
		Male PD KO: 6			
		Female SOC WT: 4			
		Female SOC KO: 4			
		Female PD WT: 6			
		Female PD KO: 5			
4	Roche	Male SOC WT: 32	Male	Play (week 4 and 7) Amphetamine challenged locomotor activity	5 7
		Male SOC HT: 12			
		Male SOC KO: 38			
		Male PD WT: 32			
		Male PD HT: 18			
		Male PD KO: 34			
5	Roche	Male SOC WT: 85	Male	Play and pharmacology	3
		Male SOC HT: 48			
		Male SOC KO: 58			

*UU*: Utrecht University; *SOC*: Socially housed during postnatal days 21-42; *PD*: Play-deprived (i.e. socially isolated during postnatal days 21-42); *WT*: wild type rats; *KO*: neurexin1α knockout rats. Week 4: PND 21-24, week 7: PND 45-49, Week 11, PND 75-77

To deprive the rats of social play, they were socially isolated during PND21-42, i.e. the period in life when social play behaviour is most abundant (Baenninger 1967; Meaney and Stewart 1981; Panksepp 1981). We have previously shown that depriving rats of social play during this period in life causes long-lasting deficits in executive functions (Baarendse et al. 2013; Bijlsma et al. 2022), and enhances the sensitivity to substance use (Baarendse et al. 2014; Lesscher et al. 2015). On PND21 ± 3 days, half of the animals were socially housed in pairs or triads. The other half of the animals were housed in pairs, but a transparent Plexiglas divider containing small holes was placed in the middle of the home cage, creating two separate but identical compartments. Through these dividers, animals were able to exchange visual, olfactory and auditory information but physically engaging in social play behaviour was prevented. On P42, the Plexiglas divider was removed and all rats were housed in pairs for the remainder of the experiment. In cohorts 1 and 3, the animals were tested for social play after 24 hrs of resocialization. In cohort 4, animals were tested after 4 days of resocialization for social play. Cohorts 1 and 3 were again tested for social behaviour and locomotor activity in adulthood (at 11 weeks of age and onwards, see below).

### Drugs

Risperidone hydrochloride (0-0.06-0.2 mg/kg) was administered 1 hour before, and methylphenidate hydrochloride (0-0.3-1.0 mg/kg) was given 30 minutes before testing for social play (see below). d-Amphetamine sulphate (0-0.25-0.5 mg/kg) was administered after 30 min habituation in the locomotor chambers. All drugs were synthetised in-house at the Roche Innovation Center Basel, and administered subcutaneously. Drug doses were carefully selected based on previous research (Vanderschuren et al. 2008; Achterberg et al. 2014; Roche in house data), and doses were calculated as salt. In view of the importance of the neck area in the expression of social play behaviour (Pellis and Pellis 1987; Siviy and Panksepp 1987), subcutaneous injections were administered in the flank.

### Social play behaviour

The procedures for social play analysis were as previously described (Vanderschuren et al. 2008). Social play behaviour in rats first emerges around weaning (PND21), peaks between PND28-35 and declines after the onset of puberty (around PND42), and low levels of play can still be observed in adult rats (Meaney and Stewart 1981; Panksepp 1981). Therefore, to capture the developmental time course of social play, nrxn1-KO and WT rats were tested for social play behaviour three times, i.e. at PND21-24 (W4, week 4), PND45-49 (W7, week 7) and PND 75-77 (W11, week 11) weeks of age. The same pairs of animals (see below) were tested after 4 and 7 weeks, and a subset of these animals was tested at 11 weeks. The effects of risperidone and methylphenidate on social play behaviour were tested in a separate cohort of animals, at PND27-28 (risperidone) and PND40-42 (methylphenidate) of age. This cohort consisted only of male rats due to the capacity of facility.

The experiments were performed in a sound attenuated chamber under red light conditions. Animals were paired with an unfamiliar partner (i.e., not a cage mate) of the same genotype, and animals in a test pair did not differ more than 10 g in body weight. The testing arena consisted of a Plexiglas cage measuring 40×40×60 cm (l × w × h), with approximately 2 cm of wood shavings covering the floor. On the two days before testing, the rats were individually habituated to the test cage for 10 min. On the test day, the animals were socially isolated for 2.5 h before testing. The test consisted of placing two animals into the test cage for 15 min. The behaviour of the animals was digitally recorded and scored live by an observer blind to genotype and housing condition. Behaviour was assessed per pair of animals or individually, depending on the experiment, using Observer 5.1 software (Noldus Information Technology BV, Wageningen, The Netherlands).

In rats, a bout of social play behaviour starts with one rat soliciting another animal to play by pouncing, i.e. touching the nape of the neck of the other animal with its snout. If the animal that is pounced upon can fully rotates to its dorsal surface, “pinning” is the result. From this position, the supine animal can initiate another play bout, by trying to gain access to the other animal’s neck. Thus, during social play, pouncing is considered an index of play solicitation, while pinning functions as a releaser of a prolonged play bout (Panksepp and Beatty 1980; Pellis and Pellis 1987; Poole and Fish 1975). Pinning and pouncing frequencies are considered the most characteristic parameters of social play behaviour in rats (Panksepp and Beatty 1980; Trezza et al. 2010). The following behaviours were therefore scored:

- Frequency of pinning: one animal lying with its dorsal surface on the floor with the other animal standing over it.
- Frequency of pouncing: one animal attempting to nose or rub the nape of the neck of the other animal.
- Duration of social exploration: one animal sniffing or grooming any part of the partner’s body
- Duration of non-social exploration: moving around in (walking or rearing) or sniffing any part of the test cage.

### Responding for social play

Behavioural testing was conducted in an operant conditioning chamber (Med Associates, Georgia, VT, USA) divided into two equally sized compartments (25 × 30 × 25 cm, l × w × h). The compartments were separated by a Plexiglas wall with 42 small holes (Ø0.5 cm) and an automated metal door in the middle. Both compartments had a metal grid floor and a Plexiglas lid which contained a house-light (2 W). One compartment was equipped with two 4.8 cm-wide retractable levers, located on opposite sides of the compartment. Above each lever was a cue light (2.5 W). One lever was designated as the active lever and the other as the inactive lever; allocation of the left or right lever as active was counterbalanced between animals, but kept constant for individual animals. Experimental events and data recording were controlled using Med-PC software (Med Associates, Georgia, VT, USA).

Operant conditioning was performed as previously described (Achterberg et al. 2016a, b). All experiments were performed under red light conditions. In this experiment, only pairs of male rats were tested due to capacity of the equipment and the facility. Rats were trained from PND24, and tested between PND37-50. A test pair consisted of one experimental animal and one unfamiliar stimulus partner of the same genotype. Rats within a test pair did not differ more than 10 g in body weight at the start of the experiment. On PND 24, test pairs were habituated to the test cage for 10 min. After the habituation session, the animals were socially isolated for 24 h/day for 5 consecutive days per week. Next, the animals received two shaping sessions on two consecutive days. During these shaping sessions, the cue light was presented, the lever retracted and the door opened whenever the experimental animal approached the active lever, after which the rats were allowed to interact for two minutes. This procedure was repeated 7 times. If an animal did not perform any active lever presses during acquisition sessions, it received an additional shaping session later that day. On the fourth day, the lever pressing sessions (20 min) commenced under a fixed ratio (FR)-1 schedule of reinforcement, under which each active lever press resulted in presentation of the cue light, retraction of both levers, and opening of the door, after which animals were allowed to freely interact for 2 min. After acquisition of the task under the FR-1 schedule (i.e., when an animal obtained at least six out of eight possible rewards on two consecutive days), a progressive ratio (PR) schedule of reinforcement was introduced. Under this schedule, the animals had to meet a response requirement on the active lever that progressively increased after every earned reward (1, 2, 4, 6, 9, 12, 15, 25, etc; Hodos 1961; Richardson and Roberts 1996). When rats met the response requirement, the cue light was illuminated, both levers retracted and the door opened for 1 min, during which the animals could freely interact. Inactive lever presses were recorded, but had no programmed consequences. A PR session continued until an animal failed to obtain a reward within 10 min. Animals received one session per day, for 5 consecutive days per week. During the other 2 days/week animals were socially housed with their original cage-mates.

After responding had stabilized, defined as obtaining at least six rewards on three consecutive days with a variation of no more than two rewards, rats were tested with a same-age same genotype unfamiliar partner. During the earned 1 min social interactions, behaviour of the rats was assessed on-line using the Observer 5.1 software (Noldus Information Technology B.V., The Netherlands). In addition to the on-line analysis, behaviour of the animals was recorded using a camera with zoom lens, video tape recorder and television monitor. Three behavioural elements were scored, i.e. frequency of pinning, frequency of pouncing, and duration of social and non-social exploration. As the accumulated time spent in play sessions varies depending on the amount of obtained rewards, frequencies and durations are expressed per minute.

### Adult social behaviour

Experiments were conducted when the animals were 11 weeks old (PND75-77), in the same boxes as described for the analysis of social play behaviour (see above). The animals were habituated to the test cages for 10 minutes on two consecutive days prior to testing. Animals were socially isolated for 24 hours before testing to enhance the motivation to engage in social interaction (Niesink and Van Ree 1982). A test pair consisted of unfamiliar animals of the same genotype, housing condition and sex and they did not differ by more than 25 grams in bodyweight. The test consisted of placing two animals into the test cage for 15 min. Behaviour was recorded and assessed as described above (see *Social play behaviour*). The following parameters were scored per test pair or individually.

Social play behaviour (see 2.3):

- Frequency of pinning
- Frequency of pouncing

Non-playful social behaviour:

- Frequency of sexual mounting
- Duration of social exploration (sniffing/grooming/licking any part of the body of the other rat, excluding the anogenital area)
- Duration of anogenital investigation (sniffing the anogenital area of the other rat)
- Duration of following or chasing (moving or running forward in the direction of or pursuing the other rat, who moves away)

Agonistic social behaviour :

- Frequency of boxing (rearing in an upright position towards the other rat, combined with both rats rapidly pushing, pawing and grabbing at each other, or one rat wrapping around the other subject)
- Frequency of kicking (one animal extends its hind paw to the other animal to keep it away from contacting the body).

Cage exploration:

Duration of non-social exploration

### Social interest: three chamber test

The experiment was conducted as described in Kentrop et al. (2018). The three-chamber arena (120 cm x 80 cm x 40 cm) consisted of a black acrylic floor and transparent acrylic walls that separate the arena into three equally sized compartments (Sociability cage for rats, Noldus Information Technology B.V., Wageningen, The Netherlands). The walls of the middle compartment contained an opening to the outer compartments that could be closed with removable slide doors. Two cylinders with a diameter of 22 cm (40 cm in height) were placed in the outer compartments to contain stimulus rats during testing. These cylinders were made of acrylic bars placed 15 mm apart to allow for close contact while preventing physical interaction. All three-chamber experiments were performed in dim light conditions (10 lux). Between tests, the arena and cylinders were cleaned with a 0.5 % v/v solution of Shureclean VK 10 (Johnson Diversey, United Kingdom) dissolved in warm water.

Animals were individually habituated to the arena without the cylinders in two 5-min habituation sessions, the week before testing. Unfamiliar, sex- and age-matched nrxn1 heterozygous rats were used as stimulus partners. The stimulus partners were habituated twice for 5 min to the cylinders, two days prior to testing. They were used for a maximum of three tests per day separated at least by one hour.

The experimental procedure consisted of three phases. Between phases, the test rat was confined in the middle compartment for approximately 1 min.

- *Phase I*: Habituation (5 min). The subject was placed in the middle compartment and the sliding doors that provided access to the outer compartments were removed. The rat was allowed to freely explore all three compartments and the two cylinders that were placed in the outer compartments. In this phase the cylinders were empty, but unfamiliar and therefore represented novel objects.
- *Phase II*: Social interest (10 min): A stimulus rat was placed in one of the cylinders, while the other cylinder remained empty. Again, both sliding doors were removed and the test rat had access to all compartments.
- *Phase III:* Social discrimination (10 min): The stimulus rat from phase II was now considered familiar. The familiar stimulus rat remained in the cylinder and a second (novel) stimulus rat was placed in the other cylinder. Both doors were opened and the test rat was allowed to freely move around the arena for 10 min.

Ethovision XT11 (Noldus Information and Technology BV, Wageningen, The Netherlands) was used to track the position of the rats in the arena. In addition to the three chamber zones, two ‘interaction’ zones, i.e. a 10 cm perimeter around the cylinders were defined. Analysed parameters included time spent in the different compartments and time spent in the interaction zones. The proportion of interaction time was calculated to assess the preference of the test rat for one of the cylinders: (time spent in the interaction zone containing the unfamiliar stimulus rat (social interest phase) or the new unfamiliar stimulus rat (social discrimination phase) / the total time spent in both interaction zones.

### Locomotor activity

Testing for horizontal locomotor activity was performed as previously described (Veeneman et al. 2011). Rats were transferred to a plastic cage (l x w x h, 50 x 33 x 40 cm) and their position was tracked five times per second for 30 min using a video-tracking system (EthoVision, Noldus Information Technology, The Netherlands). This was done twice, i.e. at 6 weeks (PND38-40, adolescence) and 11 weeks of age (PND75-77, adulthood) in a subset of the same individuals. The locomotor response to treatment with d-amphetamine was assessed in adult male rats (15 weeks of age): the animals were habituated to the apparatus (l x w x h, 40 x 40 x 30 cm, VersaMax Animal Activity Monitoring System, AccuScan Instruments, Columbus, USA) for 30 min, after which they received an injection with amphetamine (0, 0.25 or 0.5 mg/kg, s.c.), were directly put back into the apparatus and activity was recorded for an additional 60 min.

### Statistical analysis

Data was analysed using SPSS software 24 for Windows (IBM, United States) and expressed as mean + SEM. Social play and adult social behaviour were analysed using 2- or 3-way ANOVAs with genotype (WT or nrxn1-KO), housing condition (socially housed or play-deprived) and sex as between-subject factors. The effects of methylphenidate and risperidone on social play were analysed using 2-way ANOVA with genotype and treatment as between-subject factors. Responding for social play was analysed using a 2-way repeated measures ANOVA with lever (active or inactive) as the within-subject factor and genotype as the between-subject factor. The number of obtained rewards, pinning frequency, pouncing frequency and the amount of social exploration during reinforced trials were analysed using one-way ANOVA with genotype as between subject factor. Breakpoints under the PR schedule of reinforcement, i.e., the highest number of lever presses made for a single reward in a session, are derived from an escalating curve, which violates the homogeneity of variance. Therefore, breakpoints were analysed using the non-parametric Friedman test, followed by a post hoc Wilcoxon signed ranks test when appropriate. For the three-chamber test, the proportion of interaction time was analysed with a one sample Student’s t-test against 50% chance level. In addition, a between-groups analysis of the proportion of interaction time was performed using 3-way ANOVA with genotype, housing and sex as between-subject factors. Locomotor activity was expressed as distance moved in 5 min bins and was analysed using a 3-way repeated-measures ANOVA with genotype, housing and sex as between-subject factors. The effect of amphetamine on locomotion was analysed using a repeated-measures ANOVA with time as within-subject factor and genotype, housing and dose as between-subject factors. Where appropriate, post hoc analysis was performed using one-way ANOVAs and paired or unpaired Students T-tests with Bonferroni correction.

## Results

### Social play behaviour in nrxn1-KO and WT rats

**Fig. 1:**
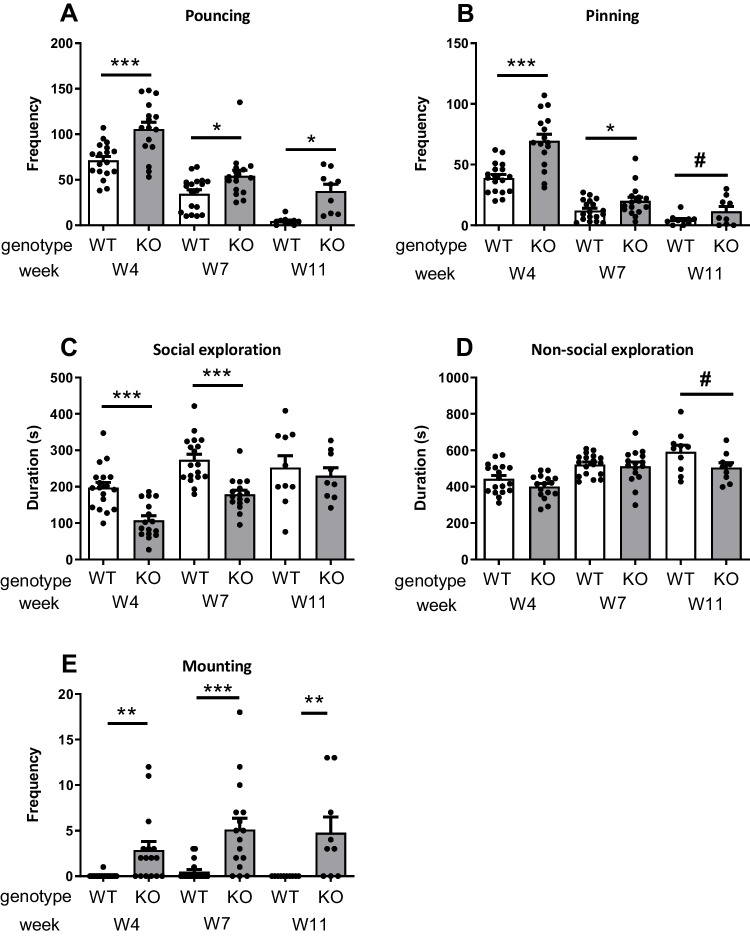
Social play behaviour in nrxn1-KO (grey bars) and WT (white bars) rats. Pouncing (**A**), pinning (**B**), social exploration (**C**), non-social exploration (**D**) and sexual mounting behaviour (**E**) at 4 weeks (W4, PND21-24), 7 weeks (W7, PND45-49) and 11 weeks of age (W11, PND75-77) in nrxn1-KO and WT rats. Data presented as mean + SEM: *p<0.05, ***p<0.001, #*p*<0.09. W4 and W7: WT: *n*=18, KO: *n*=16 pairs. W11: WT: *n*=10, KO: *n*=9 pairs

To capture the developmental time course of social play, rats were tested at 4, 7 and 11 weeks of age. Because there were no differences between male and female rats, the data were pooled. At week 4, nrxn1-KO rats pinned and pounced more than WT rats (*t*pouncing(32)=4.01, *p*<0.001 Fig. 1A; *t*pinning(32)=5.00, *p*<0.001, Fig. 1B) whereas social exploration was reduced (*t*socialexploration(32)=4.70, *p*<0.001, Fig. 1C). Non-social exploration was similar in the two genotypes (*t*nonsocialexploration(32)=1.70, *p*=0.10, Fig. 1D). Unexpectedly, the nrxn1-KO rats displayed sexual mounting behaviour during play, which was nearly absent in WT (WT, in 1 out of 18 pairs; nrxn1-KO, in 10 out of 16 pairs; *t*mounting(32)=3.18, *p*=0.003, Fig 1E).

Also at W7, the nrxn1-KO rats displayed more social play behaviour than WT rats (Fig. 1A and 1B; *t*pouncing(32)=2.60, *p*=0.01; *t*pinning(32)=2.15, *p*=0.04). Social exploration was reduced in the nrxn1-KO rats (*t*socialexploration(32)=4.97, *p*<0.001, Fig. 1C) whereas non-social exploration did not differ between nrxn1-KO and WT animals (*t*nonsocialexploration(32)=0.34, *p*=0.73, Fig. 1D). The nrxn1-KO rats also showed higher levels of sexual mounting than WT (WT, in 4 out of 18 pairs; KO, in 13 out of 16 pairs; *t*mounting(32)=3.89, *p*<0.001, Fig 1E).

At W11, the nrxn1-KO rats pounced more (*t*pouncing(17)=2.55, *p*=0.02) and tended to pin more (*t*pinning(17)=1.83, *p*=0.09, Fig. 1A,B) than WT rats whereas the amount time spent on social exploration was similar between the genotypes (*t*socialexploration(17)=0.56, *p*=0.59, Fig 1C). The time spent on non-social exploration tended to be lower in nrxn1-KO animals (*t*nonsocialexploration(17)=1.89, *p*=0.08, Fig 1D). Comparable to W4 and W7, the nrxn1-KO rats but not the WT showed sexual mounting during play (WT, in 0 out of 10 pairs; KO, in 6 out of 9 pairs; *t*mounting(32)=2.92, *p*=0.001, Fig 1E).

### Responding for social play behaviour in nrxn1-KO and WT rats

**Fig. 2:**
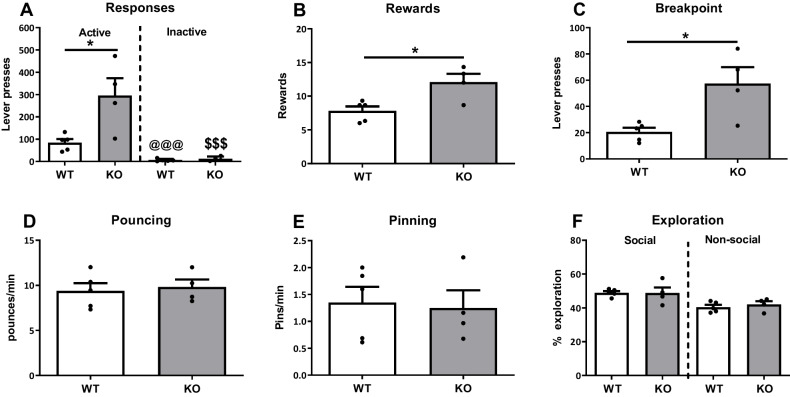
Operant responding for social play behaviour in nrxn1-KO (grey bars) and WT (white bars) rats. **A**. The amount of lever presses on the active (left panel) versus the inactive (right panel) lever. **B**. Number of rewards collected during the session. **C**. Breakpoint of responding for social play. D-E-F: Frequency of pouncing (**D**) and pinning (**E**) and percentage of time spent in social and non-social exploration (**F**) during the reinforced trials. Data are expressed as mean + SEM. *p<0.05 nrxn1-KO compared to WT, @@@ *p*<0.001 and $$$ *p*<0.001 compared to active lever-presses of the same genotype. WT: *n*=5 pairs, nrxn1-KO: *n*=4 pairs

Male rats of both genotypes learned to perform the task, as they differentiated between the levers (Flever(1,7)= 29.23, *p*=0.001), pressing the active lever significantly more than the inactive lever. The performance on the levers differed between genotypes (Fgenotype(1,7)=8.54, *p*=0.02; Flever*genotype(1,7)=9.35, *p*=0.02). When investigating performance on the active an inactive levers separately, nrxn1-KO rats made significantly more active responses than WT rats *(t(*7)=-3.14, *p*=0.02, Fig. 2A) whereas responding on the inactive lever was similar in both genotypes *(t(*7)=-0.74, *p*=0.48, Fig. 2A). Furthermore, nrxn1-KO rats collected more rewards than WT rats (*t*(7)=-3.65, *p*=0.01, Fig. 2B) and the breakpoint was higher (Z=-2.46, *p*=0.01, Fig. 2C). These results indicate a higher motivation for social play in nrxn1-KO rats.

Social play behaviour during reinforced trials, assessed as pounces and pins per minute, did not differ between the genotypes (pouncing: t(7)=0.20, *p*=0.85; pinning: *t*(7)=0.28, *p*=0.79; 2D and E). Social as well as non-social exploration in this task was also comparable in nrxn1-KO and WT rats (social exploration: *t*(7)= 1.10, *p*=0.31; non-social exploration: *t*(7)=-0.71, *p*=0.50, 2F).

### Effects of risperidone and methylphenidate on social play behaviour in nrxn1-KO and WT rats

Risperidone (0-0.06-0.2 mg/kg) affected social play behaviour in male WT and nrxn1-KO rats similarly (pouncing: Fgenotype*treatment(2,67)=0.36, *p*=0.70; pinning: Fgenotype*treatment(2,67)=0.58, *p*=0.56; social exploration: Fgenotype*treatment(2,67)=0.44, *p*=0.65) to reduce both pouncing (Ftreatment(2,67)= 14.60, *p*<0.001, *post hoc* veh vs 0.06 mg/kg: *p*=0.002; veh vs 0.2mg/kg: *p*<0.001; 0.06 vs 0.2 mg/kg: *p*=0.30, 3A) and pinning (Ftreatment(2,67)=13.45, *p*<0.001, *post hoc* veh vs 0.06 mg/kg: *p*<0.001; veh vs 0.2mg/kg: *p*<0.001; 0.06 vs 0.2 mg/kg: *p*=0.99, 3B) at both doses tested, but not social exploratory behaviour (Ftreatment(2,67)=2.57, *p*=0.08, 3C). Risperidone affected non-social exploration differently in the two genotypes (Fgenotype*treatment(2,67)=3.23, *p*=0.05; Ftreatment(2,67)=11.73, *p*<0.001). Non-social exploration was enhanced after treatment with the low but not the higher dose of risperidone in WT rats whereas it was enhanced by both doses in KO rats (*post hoc* WT: Ftreatment(1,42)=3.89, *p*=0.03, veh vs 0.06 mg/kg: *p*=0.03; veh vs 0.2mg/kg: *p*=0.18; 0.06 vs 0.2 mg/kg: *p*=0.99; KO: Ftreatment(1,25)=7.71, *p*=0.002, veh vs 0.06 mg/kg: *p*=0.08; veh vs 0.2mg/kg: p=0.002; 0.06 vs 0.2 mg/kg: *p*=0.46, 3D). Consistent with the experiments without drug treatment (see Fig. 1), male nrxn1-KO rats played more than WT animals, showing increases in both pouncing (Fgenotype(1,67)=17.72, *p*<0.001, 3A) and pinning (Fgenotype(1,67)=12,73, *p*=0.001, 3B). Nrxn1-KO rats spent less time on social and non-social exploration (social: Fgenotype(1,67)=17.77, *p*<0.001, 3C; non-social: Fgenotype(1,67)=7.53, *p*=0.008, 3D).

**Fig. 3:**
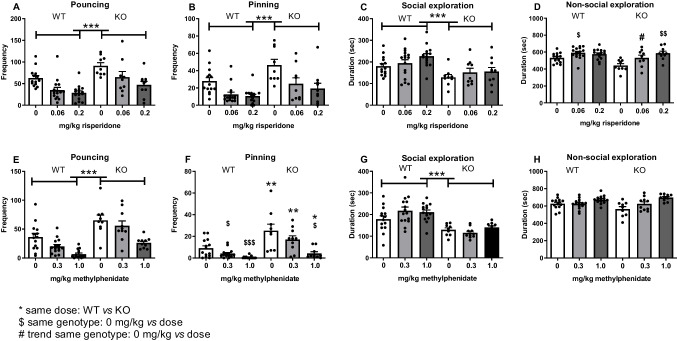
Effects of risperidone and methylphenidate on social play behaviour in male WT and nrxn1-KO rats. Risperidone (**A-D**): pouncing (**A**), pinning (**B**), social exploration (**C**) and non-social exploration (**D**) after treatment with vehicle (white bars) or risperidone 0.06 (light grey bars) or 0.2 mg/kg (dark grey bars). WT(0 mg/kg): *n*=14 pairs, WT(0.06 mg/kg): *n*=16 pairs, WT(0.2 mg/kg): *n*=15 pairs, nrxn1-KO(0 mg/kg): *n*=9 pairs, nrxn1-KO(0.06 mg/kg): *n*=9 pairs, nrxn1-KO(0.2 mg/kg): *n*=10 pairs. **Methylphenidate** (**E-H**): pouncing (**E**), pinning (**F**), social exploration (**G**) and non-social exploration (**H**) after treatment with vehicle (white bars), or methylphenidate 0.3 (light grey bars) or 1 mg/kg (dark grey bars). WT(0 mg/kg): *n*=14 pairs, WT(0.3 mg/kg): *n*=15 pairs, WT(1.0 mg/kg): *n*=15 pairs, nrxn1-KO(0 mg/kg): *n*=9 pairs, nrxn1-KO(0.3 mg/kg): *n*=10 pairs, nrxn1-KO(1.0 mg/kg): *n*=10 pairs. Data are displayed as mean + SEM. * or $*p*<0.05, **p<0.01, *** or $$$*p*<0.001, #*p*<0.08, *significant difference between WT and KO at the same dose. ^$^significant difference within one genotype between vehicle and the indicated dose. ^#^trend within one genotype between vehicle and indicated dose

Treatment with methylphenidate (0-0.3-1.0 mg/kg) suppressed pouncing (Ftreatment(2,67)=19.80, *p*<0.001, 3E) and pinning (Ftreatment(2,67)=16.53, *p*<0.001, 3F), social exploration was not affected (Ftreatment(2,67)=1.29, *p*=0.28; Fgenotype*treatment(2,67)=1.97, *p*=0.15, 3G) and non-social exploration was increased similarly in both genotypes (Fgenotype*treatment(2,67)=3.01, *p*=0.06: Ftreatment(2,67)=12.96, p<0.001; *post-hoc*: vehicle *vs* 0.3 mg/kg: *p*=0.4; vehicle *vs* 1.0 mg/kg: *p*<0.001; 0.3 *vs* 1.0 mg/kg: *p*=0.003, 3H). Again, male nrxn1-KO rats played more than WT rats (pouncing: Fgenotype(1,67)=38.98, *p*<0.001; pinning: Fgenotype(1,67)=27.50, *p*<0.001) while social exploratory behaviour was significantly reduced (Fgenotype(1,67)=45.10, *p*<0.001) and non-social exploration was unaffected (Fgenotype(1,67)=0.77, *p*=0.38). The reduction in play after methylphenidate treatment was comparable in the two genotypes for pouncing (Fgenotype*treatment(2,67)=1.16, *p*=0.32). An interaction effect of genotype and treatment was found for the frequency of pinning (Fgenotype*treatment(2,67)=3.25, *p*=0.04), methylphenidate suppressed pinning in both genotypes (*post hoc tests* WT: Ftreatment(2,43)=9.62, *p*<0.001, vehicle *vs* 0.3 mg/kg: *p*=0.04; vehicle *vs* 1.0 mg/kg: *p*<0.001; 0.3 *vs* 1.0 mg/kg: *p*=0.21, nrxn1-KO: Ftreatment(2,28)=7.02, *p*=0.004, vehicle *vs* 0.3 mg/kg: *p*=0.50; vehicle *vs* 1.0 mg/kg: *p*=0.003; 0.3 *vs* 1.0 mg/kg: *p*=0.09). After every dose, WT rats played less than nrxn1-KO rats treated with the same dose (vehicle WT *vs* nrxn1-KO: t(21)=-2.92, *p*=0.008; 0.3 mg/kg WT *vs* nrxn1-KO: t(23)=-3.59, *p*=0.005; 1.0 mg/kg WT *vs* nrxn1-KO: t(23)=-2.32, *p*=0.04) Fig. 3.

### Consequences of social play deprivation in nrxn1-KO and WT rats

**Fig. 4:**
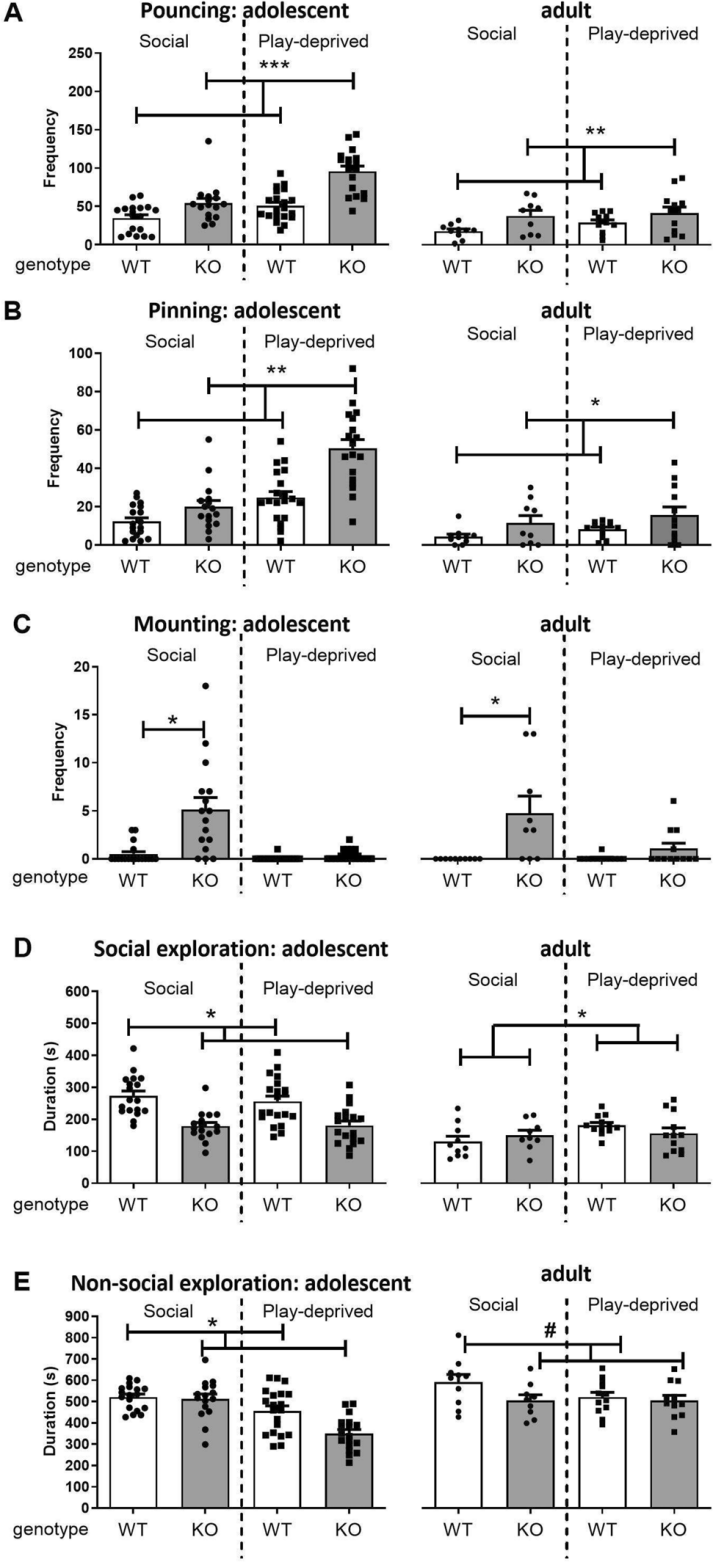
Effect of social play deprivation on social (play) behaviour in nrxn1-KO (KO) and wildtype (WT) rats, in adolescence (left set of graphs) and adulthood (right set of graphs). Pouncing (**A**), pinning (**B**), mounting (**C**), social exploration (**D**) and non-social exploration (**E**) in socially housed (left panels) nrxn1-KO (grey bars, black dots) compared to WT (white bars, black dots) rats, versus play-deprived (right panels) nrxn1-KO (grey bars, black squares) compared to WT rats (white grey bars, black squares). Data are displayed as means + SEM. ^#^*p*=0.07, *p<0.05, **p<0.01, ***p<0.001. See Table 2 for all statistics including interactions. Adolescence (7 weeks, PND45-49): socially-housed WT rats: *n*=18 pairs, socially-housed nrxn1-KO rats: *n*=16 pairs, play-deprived WT rats: *n*=16 pairs, play-deprived nrxn1-KO rats: *n*=19 pairs; Adulthood (11 weeks, PND38-40) : socially-housed WT rats: *n*=10 pairs, socially-housed nrxn1-KO rats: *n*=9 pairs, play-deprived WT rats: *n*=12 pairs, play-deprived nrxn1-KO rats: *n*=12 pairs.

To investigate whether social play deprivation would exacerbate the nrxn1-KO phenotype, rats were socially isolated from PND21-42, and social behaviour was tested during adolescence (at 7 weeks of age) or adulthood (11 weeks of age). On P42, the animals were resocialised for 4 days prior to testing. At 7 weeks of age, there were no differences between male and female rats, therefore data were pooled. nrxn1-KO rats showed more social play behaviour (Fig 4A- B, left graphs), but no modulating effect of play deprivation was observed (pouncing: Fgenotype(1,68)=36.09, *p*<0.001; Fhousing(1,68)=0.17, *p*=0.69; Fgenotype*housing(1,68)=1.90, *p*=0.17; pinning: Fgenotype(1,68)=9.34, *p*=0.003; Fhousing(1,68)=2.44, *p*=0.12; Fgenotype*housing(1,68)=0.51, *p*=0.48). Although the overall frequency was very low (1 out of 18 pairs), sexual mounting behaviour was displayed by socially housed, but not play-deprived nrxn1-KO rats (Fgenotype(1,68)=5.47, *p*=0.02; Fhousing(1,68)=3.95, *p*=0.05; Fgenotype*housing(1,68)=6.50, *p*=0.01, 4C, left graph). *Post hoc* analysis showed that compared to socially housed WT rats, socially housed nrxn1-KO rats displayed significantly more sexual mountings (Fig 4C, left panel of left graph). Social exploratory behaviour (Fig 4D, left graph) was significantly reduced in nrxn1-KO rats compared to WT rats. Play deprivation did not affect this behaviour (Fgenotype(1,68)=4.38, *p*=0.04; Fhousing(1,68)=0.41, *p*=0.52; Fgenotype*housing(1,68)= 0.005, p=0.94). Nrxn1-KO rats spent significantly less time on non-social exploratory behaviour (Fgenotype(1,68)= 6.55, *p*=0.01; Fhousing(1,68)= 0.08, *p*=0.77; Fgenotype*housing(1,68)= 0.39, *p*=0.54).

At 11 weeks of age (Fig. 4 A-E, right graphs, see Table 2 for all comparisons including statistics), adult nrxn1-KO animals played more than WT rats (Fig. 4A-B right panels). Social play deprivation did not affect play behaviour in adulthood, both pinning and pouncing were similar between housing conditions. Interestingly, whereas males and females played in equal amounts in adolescence, females made more pins in adulthood compared to males. In addition, female nrxn1-KO rats pinned more than either female WT and male nrxn1-KO rats (data not shown, see Table 2). Pouncing was similar between the sexes. nrxn-1 KO animals showed more boxing than WT animals but no effects of sex or housing condition were found. During social interactions, the amount of agonistic kicking was similar between the genotypes, sexes and housing conditions. Adult socially housed nrxn1-KO animals performed more sexual mounts compared to WT animals, while mounts in play-deprived animals were almost absent, and not different between genotypes (see Fig 4C, right graph). The number of mounts was not different between males and females.

**Table 2:** Adult social behaviour: effect of play deprivation, genotype and sex (means and statistics)

Factor		Sex, genotype, housing, mean + SEM				Main effects	Interactions	Significant interactions and post-hoc tests
Behaviour								
Pouncing	male	WT	SOC	13.00	4.51	Fgenotype(1,36)=7.92, *p*=0.008	Fgenotype*sex(1,36)=0.20, *p*=0.66; Fsex*housing(1,36)= 0.91, *p*=0.67; Fgenotype*housing(1,36)=0.68, *p*=0.42; Fgenotype*sex*housing(1,36)=0.02, *p*=0.88	-
	male	WT	PD	25.83	6.04			
	male	KO	SOC	30.83	7.17			
	male	KO	PD	35.80	14.32	Fsex(1,36)=3.25, *p*=0.08		
	female	WT	SOC	22.60	2.46			
	female	WT	PD	32.17	4.32			
	female	KO	SOC	47.50	12.53	Fhousing(1,36)=1.17, *p*=0.29		
	female	KO	PD	45.57	9.26			
Pinning	male	WT	SOC	2.80	1.24	Fgenotype(1,36)=6.36, *p*=0.02	Fsex*housing(1,36)<0.001, *p*=0.99; Fgenotype*housing(1,36)= 0.59, *p*=0.81; Fgenotype*sex*housing(1,36)=0.004, *p*=0.95	Fgenotype*sex(1,36)=4.84, *p*=0.03, post hoc tests: *t*male_WT-KO(20)=-0.40, *p*=0.70; *t*female_WT-KO(20)=-2.85, *p*=0.02; *t*WT_male-female(20)=-1.65, *p*=0.12; *t*KO_male-female(20)=-2.97, *p*=0.008
	male	WT	PD	6.83	1.87			
	male	KO	SOC	5.00	2.80			
	male	KO	PD	7.40	4.51	Fsex(1,36)=9.77, *p*=0.004		
	female	WT	SOC	6.00	2.30			
	female	WT	PD	9.67	1.20			
	female	KO	SOC	18.75	6.33			
	female	KO	PD	21.43	5.80	Fhousing(1,36)=1.40, *p*=0.25		
Boxing	male	WT	SOC	1.20	0.73	Fgenotype(1,36)=4.90, *p*=0.03	Fgenotype*sex(1,36)=0.04, *p*=0.85; Fsex*housing(1,36)=0.10, *p*=0.76; Fgenotype*housing(1,36)=0.57, *p*=0.45; Fgenotype*sex*housing(1,36)=0.64, *p*=0.42	-
	male	WT	PD	3.17	0.70			
	male	KO	SOC	10.17	6.48			
	male	KO	PD	11.80	9.60	Fsex(1,36)=2.78, *p*=0.10 Fsex(1,36)=2.78, *p*=0.10		
	female	WT	SOC	9.80	3.46			
	female	WT	PD	8.17	2.46			
	female	KO	SOC	11.50	4.05	Fhousing(1,36)= 0.64, *p*=0.43		
	female	KO	PD	21.29	5.83			
Kicking	male	WT	SOC	2.40	0.87	Fgenotype(1,36)=3.15, *p*=0.08	Fgenotype*sex(1,36)=0.15, *p*=0.70; Fsex*housing(1,36)=0.009, *p*=0.93; Fgenotype*housing(1,36)=0.004, *p*=0.95 Fgenotype*sex*housing(1,36)=0.05, *p*=0.83	-
	male	WT	PD	6.83	2.85			
	male	KO	SOC	7.00	4.56			
	male	KO	PD	13.80	10.19	Fsex(1,36)=2.93, *p*=0.10		
	female	WT	SOC	6.60	1.60			
	female	WT	PD	13.67	4.08			
	female	KO	SOC	16.25	7.86	Fhousing(1,36)= 2.09, *p*=0.16		
	female	KO	PD	22.00	7.61			
Mounting	male	WT	SOC	0.00	0.00	Fgenotype(1,36)=13.12, *p*=0.001	Fgenotype*sex(1,36)<0.001, *p*=0.99; Fsex*housing(1,36)=2.65, *p*=0.11; Fgenotype*sex*housing(1,36)=2.32, *p*=0.14	Fgenotype*housing(1,36)= 4.37, p=0.04 *Post hoc tests:* *t*soc_WT-KO(20)=-2.66, *p*=0.03; *t*pd_WT-KO(20)=-1.78, *p*=0.10; *t*WT_soc-pd(20)=-0.91, *p*=0.37; *t*KO_soc-pd(20)=2.02, *p*=0.09
	male	WT	PD	0.17	0.17			
	male	KO	SOC	3.33	2.06			
	male	KO	PD	2.60	1.03	Fsex(1,36)= 0.01, *p*=0.91 Fsex(1,36)= 0.01, *p*=0.91		
	female	WT	SOC	0.00	0.00			
	female	WT	PD	0.00	0.00			
	female	KO	SOC	5.75	2.81	Fhousing(1,36)= 3.94, *p*=0.06		
	female	KO	PD	0.00	0.00			
Social exploration	male	WT	SOC	140.22	31.73	Fgenotype(1,36)=0.01, *p*=0.98	Fgenotype*sex(1,36)=1.04, *p*=0.31; Fsex*housing(1,36)=0.82, *p*=0.37; Fgenotype*housing(1,36)=2.07, *p*=0.16; Fgenotype*sex*housing(1,36)=1.95, *p*=0.17	-
	male	WT	PD	184.29	16.21			
	male	KO	SOC	155.35	22.42			
	male	KO	PD	198.33	30.29	Fsex(1,36)=3.58, *p*=0.07 Fsex(1,36)=3.58, *p*=0.07		
	female	WT	SOC	121.17	13.58			
	female	WT	PD	179.00	8.16			
	female	KO	SOC	146.83	9.26	Fhousing(1,36)= 4.90, *p*=0.03		
	female	KO	PD	125.51	12.93			
Anogenital investigation	male	WT	SOC	14.20	3.73	Fgenotype(1,36)=0.28, *p*=0.60	Fgenotype*sex(1,36)=0.80, *p*=0.38; Fsex*housing(1,36)=2.02, p=0.16; Fgenotype*housing(1,36)=1.04, *p*=0.32; Fgenotype*sex*housing(1,36)=0.14, *p*=0.71	-
	male	WT	PD	11.83	3.82			
	male	KO	SOC	16.50	2.66			
	male	KO	PD	11.20	1.39	Fsex(1,36)=1.97, *p*=0.17 Fsex(1,36)=1.97, *p*=0.17		
	female	WT	SOC	21.80	3.72			
	female	WT	PD	14.67	3.70			
	female	KO	SOC	21.75	4.55	Fhousing(1,36)= 9.67, *p*=0.004		
	female	KO	PD	8.29	1.46			
Following behaviour	male	WT	SOC	15.00	5.55	Fgenotype(1,36)=15.24, *p*<0.001	Fgenotype*sex(1,36)=0.50, *p*=0.48; Fsex*housing(1,36)=1.04, *p*=0.31; Fgenotype*housing(1,36)=0.90, *p*=0.35; Fgenotype*sex*housing(1,36)=0.62, *p*=0.44	-
	male	WT	PD	24.83	5.76			
	male	KO	SOC	36.83	7.09			
	male	KO	PD	44.60	17.82	Fsex(1,36)=5.52, *p*=0.03		
	female	WT	SOC	27.20	3.25			
	female	WT	PD	34.00	5.49			
	female	KO	SOC	68.50	12.65	Fhousing(1,36)=0.11, *p*=0.74		
	female	KO	PD	52.71	9.56			
Non-social exploration	male	WT	SOC	573.07	40.87	Fgenotype(1,36)=3.40, *p*=0.07	Fgenotype*sex(1,36)=0.12, *p*=0.73; Fsex*housing(1,36)=0.003, *p*=0.95; Fgenotype*housing(1,36)=1.58, *p*=0.22; Fgenotype*sex*housing(1,36)=1.50, *p*=0.23	-
	male	WT	PD	534.31	37.31			
	male	KO	SOC	530.48	37.31			
	male	KO	PD	493.49	40.87	Fsex(1,36)=0.03, *p*=0.87		
	female	WT	SOC	610.59	40.87			
	female	WT	PD	506.84	37.31			
	female	KO	SOC	480.21	45.69	Fhousing(1,36)=1.69, *p*=0.20		
	female	KO	PD	514.80	34.54			

*SOC*: socially housed, *PD*: play-deprived. Social male WT: *n*=5 pairs, social male nrxn1-KO: *n*=5 pairs, play-deprived male WT: *n*=6 pairs, play-deprived male nrxn1-KO: *n*=6 pairs, social female WT: *n*=5 pairs, social female nrxn1-KO: *n*=4 pairs, play-deprived female WT: *n*=6 pairs, play-deprived female nrxn1-KO: *n*=6 pairs

Aspects of general social interest were recorded as well. Overall, play-deprived rats spent more time on social exploration compared those that were socially housed during PND21-42 (Fig 4D, right graph), but less on anogenital investigation (Table 2). These parameters were not influenced by genotype or sex. Nrxn1-KO rats spent more time following than WT rats, independent of housing, and females of both genotypes spent more time following than males. Nrxn-1 KO rats tended to spent less time on cage exploration compared to WT rats but this was independent of housing or sex (4E right graph).

### Social interest in in nrxn1-KO and WT rats

**Fig. 5:**
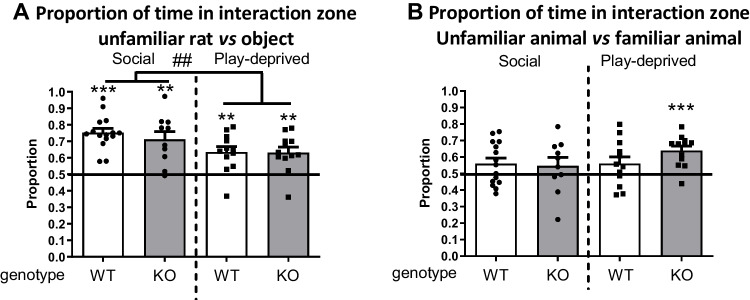
Effect of nrxn-1 KO and play deprivation on social interest and social discrimination in adult rats. **A**. Proportion of time spent in the vicinity of an unfamiliar stimulus rat versus an empty cylinder, in socially housed (social; left panels, black dots) and play deprived (right panels, black squares) WT (white bars) and nrxn1-KO (KO; grey bars) rats. **B**. Proportion of time spent in the vicinity of a novel unfamiliar rat versus the (now) familiar stimulus rat in socially housed (social; left panels, black dots) and play deprived (right panels, black squares) WT (white bars) and nrxn1-KO (KO; grey bars) rats. Data are presented as mean + SEM. *p<0.05, **p<0.01, ***p<0.001 versus 50%, ## *p*<0.01 social versus play deprived. WT social: *n*=15; nrxn1-KO social: *n*=10; WT play-deprived: *n*=12; nrxn1-KO play-deprived: *n*=12

Regardless of genotype or housing condition, the proportion of time spent investigating the unfamiliar animal compared to the empty cylinder was more than 50% in all groups (*t*WT/social(14)=9.62, *p*<0.001; *t*nrxn1-KO/social(9)=4.47, p=0.002; *t*WT/playdeprived(11)=4.04, *p*=0.002;* t*nrxn1-KO/playdeprived(11)=4.04, p=0.002), indicating a greater interest in a social stimulus compared to a non-social stimulus (Fig. 5A). The proportion of time spent on the social stimulus was not affected by nrxn1 deletion (Fgenotype(1,45)=0.41, *p*=0.53). However, socially housed animals spent a greater proportion of time close to the stimulus rat than play-deprived rats (Fhousing(1,45)=8.30, p=0.006, Fgenotype*housing(1,45)=0.31, *p*=0.58).

In the next phase of the experiment (Fig. 5B), all groups spent slightly more than 50% of their time with the novel rat compared to the familiar one, but only significantly so in play-deprived nrxn1-KO rats (*t*nrxn1-KO/playdeprived(11)=5.13, *p*<0.001). The other groups did not significantly discriminate between the familiar and unfamiliar rat (*t*WT/social(14)=1.82, *p*=0.09; *t*nrxn1-KO/social(9)=0.92, *p*=0.38; *t*WT/playdeprived(11)=1.53, *p*=0.15, Fig 5B). No significant differences in proportion of time spent investigating the novel unfamiliar rat were found between the test groups (Fgenotype(1,45)=0.72, *p*=0.40; Fhousing(1,45)=1.53, *p*=0.22; Fgenotype*housing(1,45)=1.45, *p*=0.24).

### Locomotor activity in adolescent and adult nrxn1-KO and WT rats

**Fig. 6:**
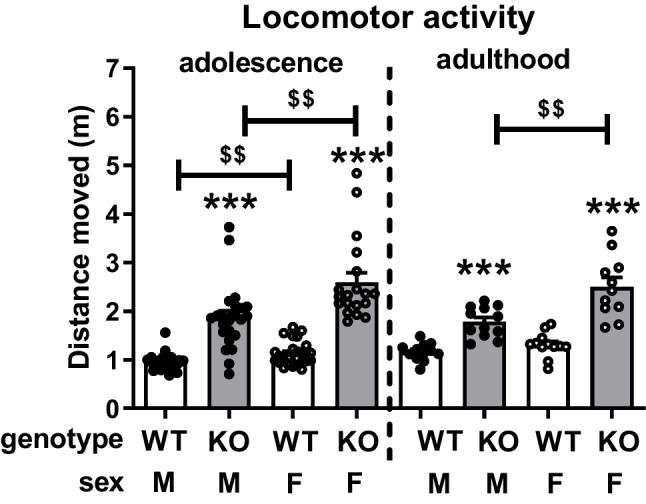
Locomotor activity in adolescent and adult nrxn1-KO and WT rats. Left panel: Locomotor activity in adolescent (6 weeks, PND38-40) male (M, black dots) and female (F, open dots) nrxn1-knock out (KO, grey bars) versus wildtype rats (WT, white bars). Right panel: locomotor activity of a subset of the same rats in adulthood (11 weeks, PND 75-77). Data are presented as mean + SEM. ***p<0.001 compared to its wildtype counterpart, $$*p*<0.01, males compared to females. Number of rats in adolescence: female WT rats: n=26, male WT rats: *n*=26, female nrxn1-KO rats: *n*=19, male nrxn1-KO rats: *n*=23; in adulthood: male WT rats: *n*=12, male nrxn1-KO rats: *n*=12, female WT rats: *n*=12, female nrxn1-KO rats: *n*=11

Locomotor activity was not affected by play deprivation, therefore data was pooled for this parameter. The rats were tested twice, during adolescence at 6 weeks (Fig. 6, left panel) and in adulthood at 11 weeks of age (Fig. 6, right panel). nrxn1-KO rats were more active than WT animals (adolescence: Fgenotype(1,90)=117.36, *p*<0.001; adulthood: Fgenotype(1,43)=66.91, *p*<0.001), and females were more active than males (adolescence: Fsex(1,90)=17.08, p<0.001; adulthood: Fsex(1,43)=15.21, *p*<0.001) and there was an interaction between genotype and sex (adolescence: Fgenotype*sex(1,90)=6.00, *p*=0.02; adulthood: Fgenotype*sex(1,43)=6.70, *p*=0.01). *Post hoc* testing revealed that in adolescence and adulthood both male and female NRXN1-KO rats were more active than WT rats. Furthermore, in adolescence and adulthood, female NRXN1-KO rats were more active than male KO rats . Female WT rats also showed more locomotor activity compared to male WT rats in adolescence but in adulthood, their locomotor activity was similar to that of males.

### Locomotor response to amphetamine in nrxn1-KO and WT rats.

**Fig. 7:**
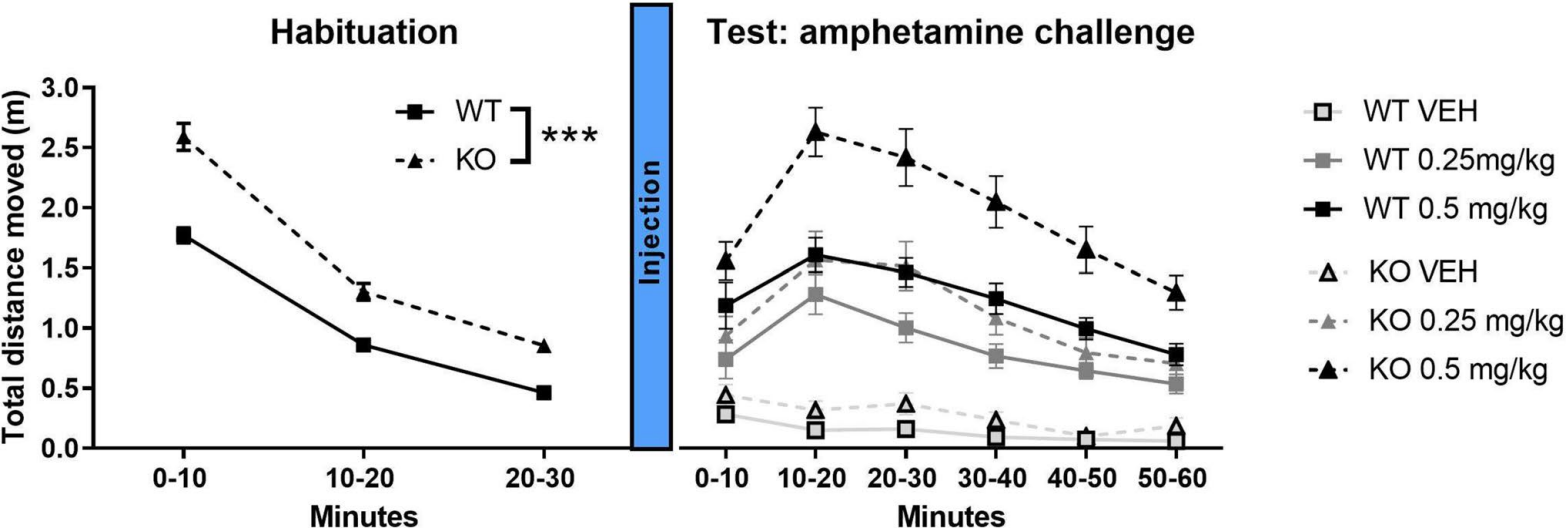
Amphetamine-induced locomotor activity. Left panel: Habituation session of 30 minutes in male nrxn1-KO (KO; black line) versus wildtype (WT, grey line) rats. Data are presented as mean ± SEM per 10 minute time bin for WT (solid lines, *n*=61), and KO (dotted lines, *n*=62) rats. ***p<0.001. Right panel: Effect of amphetamine treatment in nrxn1-KO (KO; triangles) and WT (squares) rats. Data are presented as mean ± SEM, per 10 minute time bin for vehicle (light grey lines), 0.25 mg/kg (dark grey lines), and 0.5 mg/kg amphetamine treatment (black lines). WT vehicle: *n*=20, WT 0.25 mg/kg: *n*=21, WT 0.5 mg/kg: *n*=20, nrxn1-KO vehicle: n=18, nrxn1-KO 0.25 mg/kg: *n*=23, nrxn1-KO 0.5 mg/kg: n=21

The effect of amphetamine on locomotor activity was investigated in adult males at 15 weeks of age. There was no difference between socially housed and play-deprived rats, therefore these data were pooled. Similar to the data in Fig. 6, during habituation to the test cage (Fig. 7A), nrxn1-KO rats were more active than WT rats (Fgenotype(1,121)=39.53, *p*<0.001; Ftime(1,121)=256.74, *p*<0.001, Ftime*genotype(1,121)=4.37, *p*=0.04).

After treatment with amphetamine, locomotor activity increased in both genotypes (Fdose(2,117)=66.96, *p*<0.001), whereby activity was higher in the nrxn1-KO rats (Fgenotype*dose(2,117)=3.17, *p*=0.046). *Post hoc* analysis revealed that locomotor activity was higher in nrxn1-KO rats compared to WT rats after treatment with vehicle (t(38)=-2.86, *p*=0.007) and the 0.5 mg/kg dose of amphetamine (t(39)=-3.43, *p*=0.001), but not the 0.25 mg/kg dose of amphetamine (t(42)=-1.54, *p*=0.13).

## Discussion

Proper social functioning, important throughout life, is impaired in neurodevelopmental disorders of which disruptions in the genes coding for the transsynaptic communication protein neurexin-1 is a risk factor. The purpose of this study was to assess the impact of nrxn1 deletion on the developmental trajectory of social (play) behaviour in rats, from the juvenile phase until adulthood. Furthermore, on the basis of the idea that psychiatric symptomatology is precipitated by life events in genetically predisposed individuals, we studied whether actively depriving the rats of play during their juvenile period would affect their behaviour later in life depending on the presence of this genetic vulnerability. We also investigated the effects of drugs commonly used in clinical practice to alleviate specific symptoms common to these diseases.

The main findings of these studies were that nrxn1-KO rats displayed strongly exaggerated social play behaviour throughout development compared to littermate controls, and were more motivated to engage in play. Moreover, age-inappropriate sexual mounting behaviour was observed in the nrxn1-KO rats. Nrxn1-KO rats also showed increased locomotor activity. Play deprivation subtly affected social behaviour in adulthood, but did not profoundly influence the nrxn1-KO phenotype. Treatment with risperidone and methylphenidate inhibited social play to a comparable extent in WT and nrxn1-KO rats, and an exaggerated locomotor response to amphetamine was observed in nrxn1-KO rats.

## The social behavioural phenotype of neurexin-1 KO rats

Nrxn1-KO rats showed higher levels of social play behaviour compared to their WT counterparts from their juvenile age throughout young adulthood. This robust effect was consistently observed in different cohorts of rats, in males as well as females. Both the initiation to play, pouncing, as well as the number of pins were higher in juvenile (4 weeks), and adolescent (7 weeks) nrxn1-KO rats, while social exploratory behaviour was reduced. In addition to the high levels of actual play behaviour, the motivation for social play behaviour was also higher in juvenile nrxn1-KO rats. The nrxn1-KO rats remained more playful until -at least- 11 weeks of age. In adulthood (7 months), similar levels of social approach behaviour as well as intact social discrimination were seen in the three-chamber task.

The majority of KO rats exhibited inappropriate sexual mounting behaviour towards same-sex and same-genotype social interaction partners (see also Twining et al. 2017), which was virtually absent in WT animals. This mounting behaviour started as early as week 4 of age and was still present in adulthood at 11 weeks of age. The neurobehavioural underpinnings of this aberrant behaviour remain to be identified, but it is interesting to note that inappropriate social and sexual behaviour also feature in ASD. Although sexual behaviour is an understudied aspect of ASD, in a recent review, a higher prevalence of altered sexual function was reported (Maggio et al. 2022).

The strong increase in play behaviour in the nrxn1-KO rats in our study is in contrast to the decrease in play found previously by Kight et al. (2021). Although that study used rats of the same age, and also studied same-genotype unfamiliar partners, there are also important procedural differences, which may explain the discrepancy in the obtained results. That is, the Kight et al. (2021) study scored behaviour of individual rats, averaged over 4 days of testing in a non-habituated setting. In contrast, here we looked at the play behaviour of a pair of juveniles, that were habituated to the setup on the days preceding the test. Moreover, in the present study, we isolated the animals for 2.5 hours before testing, whereas no isolation was used by Kight et al.. This isolation period is routinely used in our laboratory to evoke reliable, half-maximal levels of social play (Niesink and Van Ree 1989; Vanderschuren et al. 1995;  2008), resulting in five- to tenfold higher levels of play than in the Kight et al. study. It may therefore be that nrxn1-KO rats are more aroused by the encounter with a conspecific after the isolation. In addition, the profound hyperactivity of the nrxn1-KO rats in a novel environment (Esclassan et al. 2015; Kight et al. 2021; present study) may have interfered with the expression of social play in the Kight study, while habituation to the test environment in the present study will have mitigated this interference.

In addition to the increase in social play behaviour, the motivation for social play behaviour was also higher in the nrxn1-KO rats. The rewarding properties of social play behaviour in rats have been well-documented in place conditioning and operant conditioning setups (Calcagnetti and Schechter 1992; Trezza et al. 2011; Achterberg et al. 2016a,b, 2019). Together with the profound and consistent increase in social play observed in the nrxn1-KO rats, the heightened levels of responding for social play suggest that the rewarding value of play is enhanced in the nrxn1-KO rats. It is well-known that incentive motivation for rewards depends on dopaminergic neurotransmission in the nucleus accumbens (Salamone et al. 2012). Indeed, dopamine has been implicated in the motivation for social play (Achterberg et al. 2015) and directly stimulating dopaminergic signalling in the nucleus accumbens enhances social play behaviour (Manduca et al. 2016). Whether there is a heightened motivation for rewards in general in the nrxn1-KO rats or whether this is specific for play, remains to be studied. In any event, our observations are not consistent with a phenotype of reduced social reward, as has been suggested for ASD (Chevallier et al. 2012). The increase in social play behaviour in nrxn1-KO rats was not observed in the reinforced trials of the operant paradigm. This is because the operant conditioning setup, animals have only one minute to play per reinforced period, whereas social play expression is analysed for 15 minutes continuously. It could be that because the playful interaction is interrupted after one minute, the stimulating effects on social play are less likely to arise. The present data, together with our previous findings (Achterberg et al. 2016a,b; 2019) therefore suggest that social play expression in our operant setup may be more sensitive to manipulations that decrease social play than to those that increase this behaviour.

Alongside the increases in social play behaviour, we also observed marked locomotor hyperactivity in the nrxn1-KO rats, consistent with previous studies (Esclassan et al. 2015; Kight et al. 2021). We think that it is unlikely, however, that the enhanced social play results from mere hyperactivity, for several reasons. Thus, while play was enhanced, social exploratory behaviour was reduced, arguing against a general increase in social behaviours as a result of hyperactivity. In addition, non-social exploratory behaviour in the social test setting was not altered in the nrxn1-KO rats. Furthermore, locomotor hyperactivity and social play behaviour can be pharmacologically dissociated. For example, treatment with psychostimulant drugs in doses that evoke hyperactivity, typically results in a reduction in social play (for reviews see Vanderschuren et al. 2016; Achterberg and Vanderschuren 2023), and we have previously shown that treatment with methylphenidate in a social setting does not alter locomotion, yet suppresses social play (Vanderschuren et al. 2008). In fact, one could also argue (see above) that profound hyperactivity compromises the cognitive and motoric mechanisms necessary for the proper expression of social play. Therefore, we interpret our findings as that deletion of the nrxn1 gene results in a (socially) disinhibited phenotype, rather than that the hyperactivity of the nrxn1-KO rats non-specifically enhances any behaviour likely to occur in a given setting. This lack of inhibition in both social and non-social situations has been reported in children diagnosed with ASD (Davidson et al. 2015; Mayes et al. 2017).

## Modest behavioural consequences of social play deprivation

In the present study, we introduced deprivation of juvenile social play experience, i.e. social isolation during the three weeks in life when social play is most abundant, as a negative life event. We hypothesized that deprivation of social play would act as a second ‘hit’ on top of the genetic vulnerability conferred by the nrxn1 deletion to precipitate or exacerbate ASD- or SCZ-like aberrant behaviours. Indeed, impaired social interactions or social withdrawal during youth is thought to worsen the symptoms of ASD and SCZ (Helgeland and Torgersen 2005; Jones et al. 1994; Jordan 2003), and previous studies have shown that deprivation of social play leads to long-lasting impairments in the social, emotional and cognitive domain (Baarendse et al. 2013; Bijlsma et al. 2022; Lukkes et al. 2009a; Potegal and Einon 1989; Van den Berg et al. 1999a; Whitaker et al. 2013, for reviews see Vanderschuren and Trezza 2014; Pellis et al. 2023). Contrary to our hypothesis, however, we found no robust additional or modifying effects of play deprivation on social (play) behaviour that differed between genotypes, with one exception. That is, social play deprivation almost abolished the sexual mounting in the nrxn1-KO rats, suggesting that in this case, one aberration counteracted another one. The underlying mechanism of this fascinating effect remains to be identified, but we speculate that the reorganization of the social repertoire as a result of play deprivation (whereby animals have particular difficulties coping with challenging social situations, see below) may not allow for this inappropriate behaviour to be displayed. Social play deprivation also resulted in modest increases in social exploratory behaviour and reduced anogenital investigation in adult rats, thus slightly altering the way in which conspecifics are investigated. In the 3-chamber social approach task, play-deprived animals were attracted by a social stimulus but slightly less so than socially-housed animals. These findings on social behaviour are somewhat consistent with previous studies, that reported modest reductions in social exploratory behaviour after play deprivation (Hol et al. 1999; Van den Berg et al. 1999a, b, c; Lukkes et al. 2009a,b). Whereas in the present study we focused on the more appetitive aspects of social behaviour, previous studies have shown that in challenging social situations, such as in an encounter with an aggressive animal, play-deprived animals are less able to use the appropriate social signals to manage conflict (Van den Berg et al. 1999a, b, c; Von Frijtag et al. 2002). It may therefore be that for an interaction between play deprivation and nrxn1 deletion to become apparent, socially challenging encounters need to be assessed, rather than the relatively safe social settings used here. Taken together, play deprivation resulted in subtle social alterations but did not act synergistically to produce a specific or more severe social phenotype. Whether play deprivation leads to enhanced cognitive deficits or alterations in emotional reactivity on top of the genetic vulnerability will be the focus of future research.

## Risperidone and methylphenidate do not selectively affect play behaviour in nrxn1-KO rats, but nrxn1-KO rats are more sensitive to amphetamine

In order to put the exaggerated social play behaviour in nrxn1-KO rats into a clinical perspective, and to gauge the underlying neuropharmacological mechanisms, especially the monoamine neurotransmitter systems, we investigated the effects of risperidone and methylphenidate on social play behaviour. Risperidone is a dopamine D2/serotonin 5-HT2 receptor antagonist, that is used to treat irritability and aggression in ASD and SCZ (McCracken et al. 2002; Shea et al. 2004), and methylphenidate is a dopamine and noradrenaline reuptake inhibitor, that is used for the treatment of inattention, hyperactivity and impulsivity, that are typical of attention-deficit hyperactivity disorder (ADHD; Biederman and Faraone 2005; Feldman and Reiff 2014), but that also occurs in ASD (Murray 2010; Krakowski et al. 2020). In addition, both risperidone and methylphenidate have been shown to alleviate social deficits in SCZ and ASD animal models as well as patients (Hara et al. 2016; Kamińska and Rogóż, 2015; Wang et al. 2007; Smith et al. 1998; Harvey et al. 2006). To investigate the sensitivity to psychostimulant drugs, we assessed the hyperactivity evoked by amphetamine (that stimulates the release of dopamine, noradrenaline and serotonin), that is also used for the treatment of ADHD (Biederman and Faraone 2005; Feldman and Reiff 2014) but exacerbates positive symptoms of SCZ (Meltzer and Stahl 1976; Kapur 2003).

Risperidone reduced social play behaviour to an equal extent in both genotypes. This antipsychotic is thought to exert its effects through antagonism of dopamine D2 and serotonin 5-HT2 receptors, but it also antagonizes noradrenergic alpha-1 and -2 and histaminergic H1 receptors. Treatment with dopamine D2 and alpha-1 receptor antagonists has previously been shown to inhibit social play, whereas treatment with an alpha-2 adrenoceptor antagonist actually enhances it (Siviy et al. 1994; -1996). The effects of serotonin 5-HT2 and histamine H1 receptor antagonism on social play remain to be properly investigated. Thus, the play-suppressant effect of risperidone likely depends on blockade of dopamine D2 receptors, alpha-1 adrenoceptors, or both, whereby the activity of these receptor systems does not differ between WT and nrxn1-KO rats. That said, the possibility that risperidone suppresses social play behaviour through distinct pharmacological mechanisms in the two genotypes can as yet not be excluded. From a clinical point of view, the comparable sensitivity to risperidone in WT and nrxn1-KO rats argues against the possibility that the increased play behaviour in nrxn1-KO rats reflects heightened levels of aggression, which is suggested by the increased levels of boxing, kicking and chasing observed in young adult nrxn1-KO rats – an explanation that would also be unlikely given the structural differences in social play and aggression in rats (e.g. Pellis and Pellis 1987). Moreover, the fact that risperidone did not affect social exploration and increases non-social exploration indicates that at the doses tested, the reduction in social play cannot be explained by general sedative effect of D2 receptor blockade.

Methylphenidate, the most prescribed drug for the treatment of ADHD, reduced social play behaviour in both genotypes without affecting social and non-social exploration. This specific reduction in social play after methylphenidate treatment is consistent with previous findings (Beatty et al. 1982; Vanderschuren et al. 2008), and we have demonstrated that methylphenidate inhibits social play behaviour through indirect stimulation of alpha-2 adrenoceptors (Vanderschuren et al. 2008; Achterberg et al. 2016a, b). The comparable sensitivity to methylphenidate in WT and nrxn1-KO rats indicates that the increase in social play is not the result of altered alpha-2 noradrenergic signalling in nrxn1-KO rats. We have previously argued that the play-suppressant properties of methylphenidate are due to increased behavioural inhibition, whereby energetic behaviours that result in diminished attention for the environment are reduced (Vanderschuren et al. 2008). That said, the subsequent findings that methylphenidate reduces social play through both prefrontal and subcortical limbic structures implies that alongside increased – prefrontal – inhibition, methylphenidate may also alter the – subcortically mediated – emotional properties of social play (Achterberg et al. 2015). A disinhibited phenotype could therefore underlie the exaggerated social play in nrxn1-KO rats, but it is not one that results from altered noradrenergic neurotransmission.

We also assessed the sensitivity to the psychomotor effects of amphetamine. In this experiment, nrxn1-KO rats were hyperactive, as was found previously (Esclassan et al. 2015; Kight et al. 2021), and although amphetamine-induced hyperactivity was observed in both WT and nrxn1-KO animals, the latter showed a heightened response to the highest dose of amphetamine tested. Given that both novelty-induced locomotor activity (Hooks and Kalivas 1994) and the hyperactivity evoked by amphetamine depend on nucleus accumbens dopamine neurotransmission (Creese and Iversen 1975; Pijnenburg et al. 1975), these findings suggest that nucleus accumbens dopaminergic neurotransmission is hypersensitive in nrxn1-KO rats. Increased sensitivity of mesolimbic dopamine has been implicated in SCZ (Kapur 2003), and it has also been observed in the methylazoxymethanol acetate model of SCZ (e.g. Gomes et al. 2014). Thus, the increased sensitivity to amphetamine in nrxn1-KO rats is consistent with an SCZ-like phenotype. Conversely, the lack of a corrective effect of amphetamine treatment on the nrxn1-KO phenotype in the direction of WT suggests that this overactive locomotor phenotype might not be ADHD-like hyperactivity.

## Conclusion

In the present study, we demonstrate an aberrant social phenotype of nrxn1-KO rats, apparent as exaggerated social play, increased motivation for play and inappropriate sexual mounting. In contrast to our hypothesis, nrxn1-KO rats did not display reduced social interaction or social withdrawal reminiscent of ASD or SCZ symptomatology, and the intended ‘second hit’ of social play deprivation had no major consequences for social behaviour. Nrxn1-KO rats displayed an overall increase in general activity, increased responsiveness to amphetamine, but risperidone and methylphenidate did not selectively alter the social phenotype of nrxn1-KO rats. Together, these findings suggest that deletion of the nrxn1 gene results in a disinhibited phenotype, in both a social and a non-social setting. The neurexin-1 knockout rat could therefore be used as a model for inappropriate or disinhibited social behaviour seen in childhood mental disorders.
